# B7-H3: a consistent marker in metastatic colorectal cancer with potential for targeted treatment

**DOI:** 10.3389/pore.2025.1612186

**Published:** 2025-08-13

**Authors:** Julia M. Ott, Verena Gassenmaier, Michael Bitzer, Christian M. Schürch, Jonas S. Heitmann, Ilona Hagelstein

**Affiliations:** ^1^ Clinical Collaboration Unit Translational Immunology, Department of Internal Medicine, University Hospital Tuebingen, Tuebingen, Germany; ^2^ Department of Internal Medicine I (Gastroenterology, Gastrointestinal Oncology, Hepatology, Infectious Diseases and Geriatrics), University Hospital Tuebingen, Tuebingen, Germany; ^3^ Cluster of Excellence iFIT (EXC 2180) “Image–Guided and Functionally Instructed Tumor Therapies”, Eberhard Karls University Tuebingen, Tuebingen, Germany; ^4^ Department of Pathology and Neuropathology, University Hospital and Comprehensive Cancer Center Tuebingen, Tuebingen, Germany; ^5^ Center for Personalized Medicine, Eberhard Karls University Tuebingen, Tuebingen, Germany; ^6^ German Cancer Consortium (DKTK), Partner Site Tuebingen, a Partnership Between DKFZ and University Hospital Tuebingen, Tuebingen, Germany

**Keywords:** colorectal cancer, immunotherapy, targeted therapy, B7-H3, CD276, bispecific antibody

## Abstract

Colorectal cancer (CRC) remains a leading cause of cancer-related morbidity and mortality worldwide. Despite advances in various treatment approaches, outcomes for patients with metastatic CRC (mCRC) remain poor, and treatment-associated side effects significantly impact quality of life. While immunotherapy has shown promise in certain malignancies, its efficacy in CRC is limited to a minority of patients, highlighting the urgent need for novel therapeutic targets to improve treatment efficacy while minimizing off-target effects. B7-H3 (CD276) has emerged as a promising immunotherapeutic target due to its selective expression on tumor cells and neovasculature, with minimal presence in healthy tissues. A novel IgG-based bispecific antibody targeting B7-H3 and CD3, CC-3, has demonstrated strong preclinical efficacy in stimulating T cell-mediated antitumor responses and is currently being evaluated in a first-in-human trial including patients with mCRC (NCT05999396). In this study, we investigated B7-H3 expression in a cohort of n = 55 mCRC patients and assessed its correlation with demographic, pathological, and molecular factors, as well as clinical outcomes. Additionally, to evaluate the stability of B7-H3 expression over time, we analyzed sequential biopsies from metastatic lesions from n = 7 patients at subsequent time points. Our findings demonstrate that B7-H3 is consistently overexpressed in mCRC, independent of demographic factors, primary tumor localization (right vs. left colon), common molecular and genetic alterations (HER2, MSI, KRAS, NRAS, BRAF, PIK3CA, p53), and serum tumor markers. Longitudinal analysis showed that B7-H3 expression was comparable or increased over time in sequential metastatic specimens. No significant association was observed between B7-H3 expression and overall survival or progression-free survival, and prior chemotherapy treatment did not influence B7-H3 expression levels. In conclusion, B7-H3 is stably and ubiquitously expressed in mCRC, reinforcing its potential as a robust target for immunotherapeutic strategies, including bispecific antibodies. The lack of variability across patient subgroups suggests that routine pre-treatment assessment of B7-H3 may not be necessary. These findings provide a strong rationale for the continued clinical evaluation of B7-H3-targeted therapies, such as CC-3 (NCT05999396), in mCRC patients.

## Introduction

Colorectal cancer (CRC) is the third most commonly diagnosed malignancy and the second leading cause of cancer-related death worldwide [1]. Despite advancements in early detection, surgical intervention, systemic treatment including chemotherapy and monoclonal antibodies and radiotherapy, the prognosis for patients with advanced and particularly metastasized CRC (mCRC) remains poor [2]. While immune checkpoint inhibitors (ICIs) have shown impressive results in the treatment of various solid tumors [3], their efficacy in CRC is largely restricted to tumors with high microsatellite instability (MSI-H) or defective DNA mismatch repair (dMMR), representing only 15% of cases [4] and dropping to 4% in the metastatic setting [5, 6]. This highlights the urgent need for novel therapeutic targets to improve treatment options for the majority of mCRC patients and a deeper understanding of the immune mechanisms underlying CRC progression and therapy resistance.

One such mechanism involves the immune checkpoint protein B7-H3, a member of the B7 family. Although its precise role in antitumor immunity remains debated, B7-H3 has been implicated in tumor proliferation, angiogenesis, immune evasion, metastasis, and drug resistance [7–10]. B7-H3 is highly expressed on cancer cells and tumor vasculature in multiple malignancies, including CRC, while showing limited expression in normal tissues [11, 12]. Interestingly, the expression of B7-H3 has been associated with worse patient outcomes in many solid malignancies, including CRC [13].

Emerging strategies, such as bispecific antibodies (bsAbs) targeting CD3 and tumor-associated antigens or chimeric antigen receptor (CAR) T cells, have demonstrated success in hematologic malignancies and are meanwhile well established in this field, while their application in solid tumors has encountered greater challenges [14, 15]. Nevertheless, a few bsAbs targeting solid tumors have recently received regulatory approval. For example, zenocutuzumab, a bispecific HER2xHER3 antibody, has been approved by the U.S. Food and Drug Administration (FDA) for the treatment of Neuregulin 1-positive (NRG1+) non-small cell lung cancer and pancreatic ductal adenocarcinoma [16]. For CRC no bsAb therapy is currently approved; however, a bispecific carcinoembryonic antigen (CEA) × CD3 antibody was evaluated in a phase I clinical trial (NCT02291614), with 28% of patients achieving stable disease as their best response [17]. A key challenge in solid tumors is the limited infiltration of immune cells into the tumor microenvironment, where B7-H3 may serve as an ideal target due to its expression on tumor cells and vasculature [12, 18]. Based on this hypothesis we developed a B7-H3xCD3-bsAb, currently being evaluated in a first-in-human clinical trial for patients with mCRC (NCT05999396) [19].

To better define the clinical relevance of B7-H3 in mCRC, its expression in a cohort of 55 patients with distant metastases either in the liver or lung was analyzed and the association with various clinical, pathological, and molecular parameters was analyzed. Additionally, the prognostic impact of B7-H3 expression on progression-free survival (PFS) and overall survival (OS) was assessed. A key novelty of this study is the longitudinal evaluation of B7-H3 expression in sequential tumor samples, providing insights into its stability over time.

## Materials and methods

### Patient samples

Freshly sectioned tumor samples were formalin-fixed and paraffin-embedded (FFPE). Tumor samples were obtained from the biobank of the Institute of General and Molecular Pathology and Pathological Anatomy Tuebingen. The samples used in this study were obtained from 55 patients with mCRC, who were treated at the University Hospital of Tuebingen between 2010 and 2023. At the time of sample acquisition, all patients had reached the metastatic stage (stage IV according to the Union Internationale Contre le Cancer (UICC)), bearing distant metastases either in the liver or lung. The specimens included tissue from primary colorectal tumors (n = 33), liver metastases (n = 12), lung metastases (n = 9), and peritoneal metastases (n = 1).

Additionally, we analyzed sequential samples from a randomly selected subset of n = 7 patients.

All relevant clinical data were extracted from the digital patient records. Data on PFS and OS were available for 47 and 49 patients, respectively, and were included in the corresponding analyses. Patient follow-up was conducted until death or the last documented clinical contact.

### Tissue microarray (TMA) construction

To identify suitable patient tissue specimens, hematoxylin and eosin (H&E) stained histological sections were examined microscopically to confirm the presence of tumor tissue, as verified by a board-certified pathologist. The tissue samples included both resection specimens and core needle biopsies. Two tissue cores (if possible) with a diameter of 1.0 mm were then extracted from corresponding FFPE blocks to construct a tissue microarray (TMA). To ensure representativeness, we aimed to sample tumor areas from both the center and the invasive front whenever possible, making the TMA heterogeneous by design. In certain cases, however, only liver biopsies or limited tissue material were available, thus precluding the extraction of two cores. In total, 124 cores including 2 control cores (healthy liver) were placed on a single TMA block.

### Immunohistochemistry

Immunohistochemical staining was performed on FFPE tissue sections (2.5 µm) mounted on coated TOMO slides. Slides were deparaffinized at 72°C using EZ-Prep solution (Roche, Basel, Switzerland), followed by heat-induced epitope retrieval at 100°C for 64 min in a slightly basic Tris-based buffer (CC1, Ventana Roche). After cooling, slides were incubated with the primary antibody RBT-CD276 (Medac/Bio SB), a rabbit polyclonal antibody specific for B7-H3 (CD276), at 37°C for 32 min. Signal detection was performed using the OptiView DAB Detection Kit (Ventana Roche). This detection system includes incubation with a hapten-based amplification complex consisting of a cocktail of polyclonal secondary antibodies (goat anti-rabbit IgG, goat anti-mouse IgG, and goat anti-rabbit IgM), followed by an HRP-conjugated multimer for 8 min. Visualization was achieved using 3,3′-diaminobenzidine (DAB) chromogen for 8 min. Sections were then counterstained with hematoxylin (20 min), blued (8 min), washed in distilled water, dehydrated through graded alcohols, cleared in xylene, and mounted with Cytoseal (Epredia, Portsmouth, NH).

### Assessment of B7-H3 expression

Stained slides were scanned using a Pannoramic MIDI Scanner (3DHISTECH, Budapest, Hungary), and digital image analysis was performed using the SlideViewer software (3DHISTECH) for optical examination. The staining intensities in both tumor tissue and stroma were assessed by an advanced pathology resident (VG) supervised by a board-certified pathologist (CMS) using an established scale from 0 to 3: 0 (no staining), 1 (weak staining), 2 (moderate staining), and 3 (strong staining) [20]. Two replicate samples of each patient were averaged, and the Histo-Score (H-Score) was calculated using the following formula [21]:

H-Score = (% of unstained tumor cells × 0) + (% of weakly stained tumor cells × 1) + (% of moderately stained tumor cells × 2) + (% of strongly stained tumor cells × 3).

For further analysis, the patients were divided into three equally sized groups based on H-Score levels, with the following ranges:

• Tertile 1: Low H-Score (0–190)
• Tertile 2: Medium H-Score (190–285)
• Tertile 3: High H-Score (285–300)

In cases where patients had identical H-Score values at the boundary between two tertiles (190 or 285), these patients were randomly assigned to one of the adjacent groups. This randomization strategy was applied to ensure equal group sizes for subsequent analyses.

### Statistical analysis

Continuous data were assessed for distribution. For testing normality, the Shapiro-Wilk test was used. Group differences were analyzed using ANOVA for normally distributed data and the Kruskal-Wallis test for non-normally distributed data. Post hoc pairwise Dunn tests with Benjamini-Hochberg correction were applied where appropriate. Fisher’s exact test was used to compare categorical variables between groups. Correlations were evaluated using Spearman’s rank correlation.

Survival analyses were performed using the Kaplan-Meier method. OS was defined as the time from diagnosis to death or to the last contact for censored patients. Patients who were alive at the time of analysis were censored, and deceased patients were considered events. Kaplan-Meier curves were generated using the R survival package, and differences between survival curves were assessed using the log-rank test.

PFS was defined as the time from diagnosis to documented disease progression or death. Patients with missing longitudinal data were excluded. Since all patients with available follow-up data experienced a progression event, no censoring was applied. A log-rank test was used to compare PFS between groups.

All statistical tests were conducted at a significance level of p < 0.05.

All statistical analyses and figure generation were conducted in R Studio (R version 4.4.1).

## Results

### Clinical characteristics of the patient cohort

For the assessment of B7-H3 expression, tumor samples from 55 patients were analyzed. The mean age of the patients was 60.1 years. The male-to-female ratio was 1.29:1. At the time of analysis, 85.7% of patients were deceased, with a median OS of 35 months and a median PFS of 12 months. The majority of patients (62.3%) presented with T-stage T3, followed by T4 (28.3%), indicating the depth of tumor invasion. Regarding lymph node involvement, 44.4% had N-stage 1, and 27.8% had N-stage 2. Most patients (78.0%) exhibited a histopathological tumor grade (G) of 2. At the time of the initial biopsy, all patients (100%) had already reached the metastatic stage, and at the time of diagnosis, 87.3% had metastases. A higher proportion of left sided primary tumors (63.0%) was observed compared to right sided tumors (37.0%). The patients underwent a median of 3 lines of chemotherapy, and 47.3% had received chemotherapy prior to the initial biopsy used for our TMA. With respect to genetic and molecular markers, 11.1% of patients exhibited tumors with MSI (MSI-high or MSI-low), 61.4% harbored a KRAS-, 2.9% a NRAS-, 8.8% a BRAF-, 19.1% a PIK3CA- and 80.0% a p53 mutation. HER2 expression scores were available for only 15 patients, of whom 4 showed a score of 2, and 2 showed a score of 1. The mean serum CEA level at diagnosis was 246.4 μg/L, while the mean serum CA19-9 (carbohydrate antigen 19-9) level was 1285.6 kU/L (Table 1).

**TABLE 1:** Clinical characteristics of the patient cohort.

Group	Female (n = 24)	Male (n = 31)	All cases (n = 55)
Demographic information			
Age at diagnosis in years (Mean ± SD)	58.0 ± 14.1	61.7 ± 15.4	60.1 ± 14.9
Age at biopsy in years (Mean ± SD)	58.8 ± 14.1	63.2 ± 15.3	61.3 ± 14.8
Deceased at time of analysis (n = 49)	20 (90.9%)	22 (81.5%)	42 (85.7%)
PFS in months (Median (IQR)) (n = 47)	12 (10.0–16.0)	11.5 (5.3–17.5)	12 (7.5–16.0)
OS in months (Median (IQR)) (n = 49)	29 (21.8–45.5)	39 (18.0–80.0)	35 (21.0–50.0)
Staging			
Depth of invasion (T) at diagnosis (n = 53)			
T1	0 (0.0%)	1 (3.2%)	1 (1.9%)
T2	1 (4.5%)	3 (9.7%)	4 (7.6%)
T3	13 (59.1%)	20 (64.5%)	33 (62.3%)
T4	8 (36.4%)	7 (22.6%)	15 (28.3%)
Nodal metastasis (N) at diagnosis (n = 54)			
N0	7 (30.4%)	8 (25.8%)	15 (27.8%)
N1	12 (52.2%)	12 (38.7%)	24 (44.4%)
N2	4 (17.4%)	11 (35.5%)	15 (27.8%)
Lymphatic invasion (L) at diagnosis (n = 50)			
L0	12 (54.6%)	12 (42.9%)	24 (48.0%)
L1	10 (45.5%)	16 (57.1%)	26 (52.0%)
Grading (G) at diagnosis (n = 50)			
G1	0 (0.0%)	1 (3.7%)	1 (2.0%)
G2	20 (87.0%)	19 (70.4%)	c39 (78.0%)
G3	3 (13.0%)	7 (25.9%)	10 (20.0%)
Metastases at diagnosis (%)	21 (87.5%)	27 (87.1%)	48 (87.3%)
Metastases at biopsy (%)	24 (100%)	31 (100%)	55 (100%)
Tumor localization (n = 54)			
Left sided (%)	15 (62.5%)	19 (63.3%)	34 (63.0%)
Right sided (%)	9 (37.5%)	11 (36.7%)	20 (37.0%)
Chemotherapy			
Number of chemotherapy lines (Median (IQR)) (n = 41)	4.0 (3.0–5.0)	2.5 (1.0–4.3)	3.0 (2.0–5.0)
Chemotherapy before biopsy (%)	13 (54.2%)	13 (42.0%)	26 (47.3%)
Genetic and Molecular Markers			
Microsatellite Instability (%) (n = 45)	2 (10.5%)	3 (11.5%)	5 (11.1%)
KRAS mutation (%) (n = 44)	12 (63.2%)	15 (60.0%)	27 (61.4%)
NRAS mutation (%) (n = 35)	1 (5.9%)	0 (0.0%)	1 (2.9%)
BRAF mutation (%) (n = 34)	1 (6.2%)	2 (12.5%)	3 (8.8%)
HER-2 expression Score 0/1/2 (n = 15)	5/1/1	4/1/3	9/2/4
PIK3CA mutation (%) (n = 21)	3 (27.3%)	1 (10.0%)	4 (19.1%)
P53 mutation (%) (n = 25)	10 (76.9%)	10 (83.3%)	20 (80.0%)
Serum Tumor Markers			
CEA at diagnosis in µg/L (Mean ± SD) (n = 35)	483.8 ± 965.2	338.3 ± 769.2	246.4 ± 543.5
CA19-9 at diagnosis in kU/L (Mean ± SD) (n = 29)	2629.5 ± 5252.7	206.1 ± 447.9	1285.6 ± 3666.9

Abbreviations: SD: standard deviation; IQR: interquartile range; OS: overall survival; PFS: progression-free survival; T: depth of invasion of the primary tumor; N: nodal metastasis; L: lymphatic invasion. For cases where data were unavailable, the number of available cases is indicated in parentheses.

### Established risk factors and patient outcomes

To demonstrate that the studied cohort represents a typical patient population with CRC, an analysis of established risk factors was conducted, examining their influence on PFS and OS.

A higher T-stage, according to the TNM classification, reflects deeper tumor invasion and is associated with worse outcomes [22]. In our cohort, a Logrank test revealed no significant difference in PFS between T-stage groups (p = 0.22), nor in OS (p = 0.13) (Supplementary Figure S1A).

Nodal metastases (N-stage) are a well-established adverse prognostic factor in colorectal cancer [23]. In our cohort, no significant association between N-stage and PFS was observed (p = 0.11). For OS, however, the difference between groups was significant (p = 0.01) (Supplementary Figure S1B).

The presence of lymphatic invasion (L1 according to the TNM classification) is a known independent adverse prognostic factor in colorectal carcinoma patients with liver metastases [24]. While no such difference was observed for PFS (p = 0.32), OS differed significantly between patients with and without lymphatic invasion (p = 0.02) (Supplementary Figure S1C).

A poor histological grading is known to be an independent prognostic factor in colorectal cancer [25]. In our cohort, PFS differed significantly according to tumor grading (p = 0.01), indicating a relevant impact of tumor differentiation. For OS, a trend toward worse outcomes with poor grading was observed (p = 0.14) (Supplementary Figure S2A).

The presence of metastases at diagnosis is known to be an adverse prognostic factor in colorectal cancer patients [26]. While no significant difference was observed for PFS in our analysis (p = 0.09), patients presenting with metastases at initial diagnosis of CRC had significantly worse OS (p = 0.01) (Supplementary Figure S2B).

Several studies have highlighted the prognostic significance of tumor laterality in colorectal cancer, with right-sided tumors being associated with a worse prognosis compared to left-sided tumors [27]. In our cohort, right-sided tumors were significantly associated with poorer PFS (p = 0.05) and OS (p < 0.001) (Supplementary Figure S2C).

### Expression of B7-H3 in metastatic colorectal cancer patients

**FIGURE 1:**
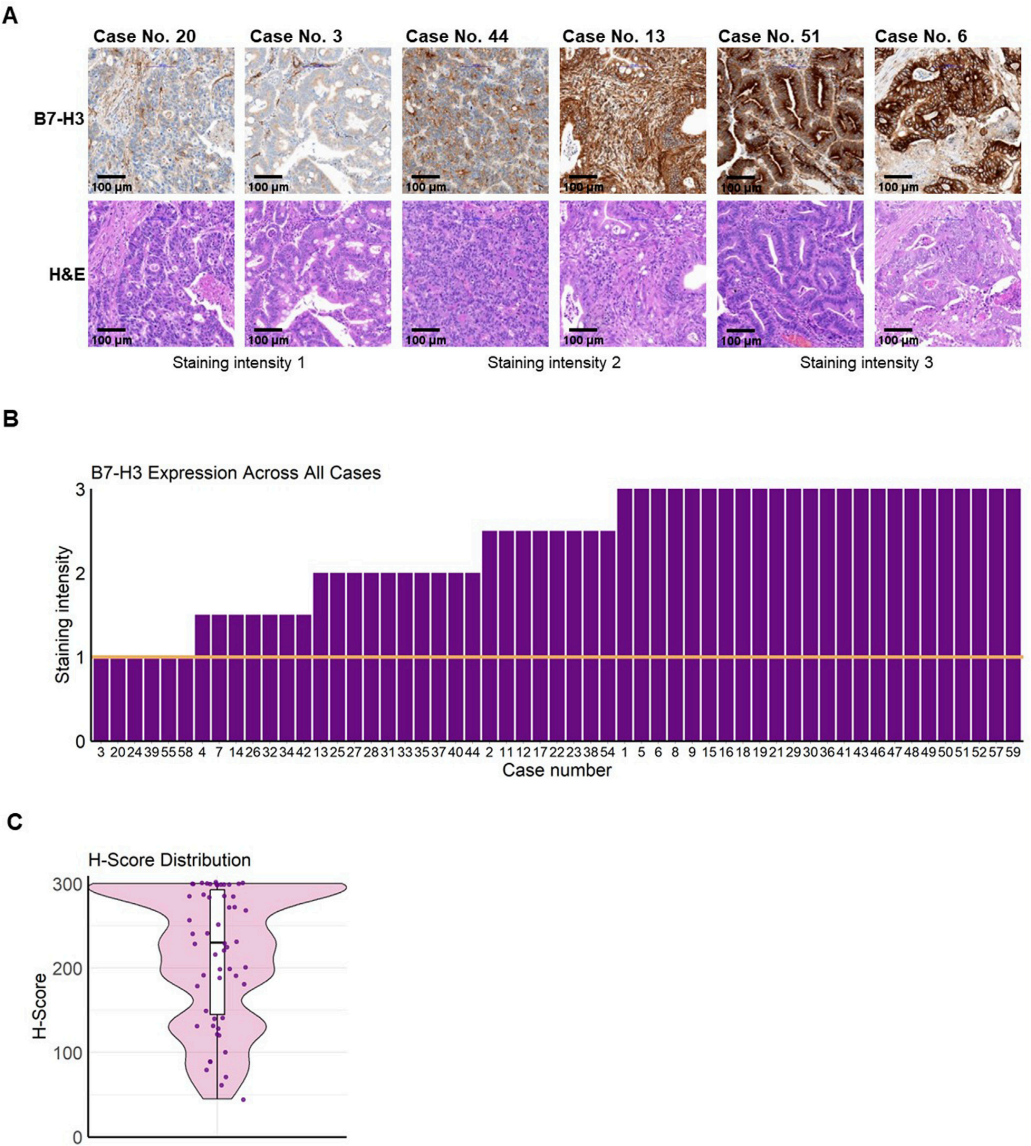
B7-H3 expression and distribution in mCRC. Freshly cut colorectal carcinoma tissues were formalin-fixed and paraffin-embedded and a tissue microarray (TMA) was created. TMA sections were analyzed for B7-H3 expression by immunohistochemistry. Staining intensity was graded under the supervision of a board-certified pathologist using the following scale: 0 (no staining), 1 (weak staining), 2 (moderate staining), and 3 (strong staining). **(A)** Representative images of tumor tissue sections are shown with the corresponding staining intensities (upper panels) and H&E stains (lower panels). The first two images show B7-H3 staining intensity of 1, the next two show intensity 2, and the final two depict intensity 3. Bar scale 100 µm. **(B)** Bar plot visualizing the mean B7-H3 expression score across all cases. The y-axis represents the B7-H3 staining intensity (ranging from 0 to 3), and the x-axis represents the individual cases. A threshold for positive expression was set at a score greater than 1, indicated by the orange line. **(C)** Violin plot showing the distribution of H-Scores, representing B7-H3 expression (H-Score = (% of unstained tumor cells × 0) + (% of weakly stained tumor cells × 1) + (% of moderately stained tumor cells × 2) + (% of strongly stained tumor cells × 3)). The black line represents the median, and the white bars indicate the interquartile range (IQR).

B7-H3 was observed as membranous staining on colorectal cancer cells in TMA sections from mCRC patients (Supplementary Figure 3A; Figure 1A). All replicates of tumor tissues from the n = 55 mCRC patients analyzed showed B7-H3 expression with a staining score of ≥1 (Figure 1B). A strong averaged staining intensity (staining score = 3) was observed in 43.6%, an intermediate averaged staining intensity (staining score = 2) was observed in 18.2%, and 10.9% of the cases were stained with a weak intensity (staining score = 1). Additionally, 14.6% of patients had a mean staining score of 2.5, and 12.7% had a score of 1.5 (Table 2). Notably, none of the analyzed mCRC patient tissues was negative for B7-H3. The mean percentage of positively stained cells was 95.0%, ranging from a minimum of 45.0% to a maximum of 100% B7-H3-positive cells. H-scores were predominantly elevated, with 100% of the metastatic colorectal cancer cases analyzed having an H-score ≥45. The median H-score was 230, with a range from 45 to 300 and an interquartile range (IQR) of 147.5 (Figure 1C). To further assess the consistency and pattern of B7-H3 expression beyond TMA sampling, whole slide imaging (WSI) was performed on tissue sections from three mCRC patient tumors. These analyses confirmed uniform B7-H3 expression across both primary tumors and metastatic lesions (Supplementary Figure S3B).

**TABLE 2:** Expression intensity of B7-H3 for mCRC patients by immunohistochemistry.

Staining intensity				
3 (%)	2.5 (%)	2 (%)	1.5 (%)	1 (%)
43.6	14.6	18.2	12.7	10.9

### Clinical and pathological parameters and B7-H3 H-Score

**FIGURE 2:**
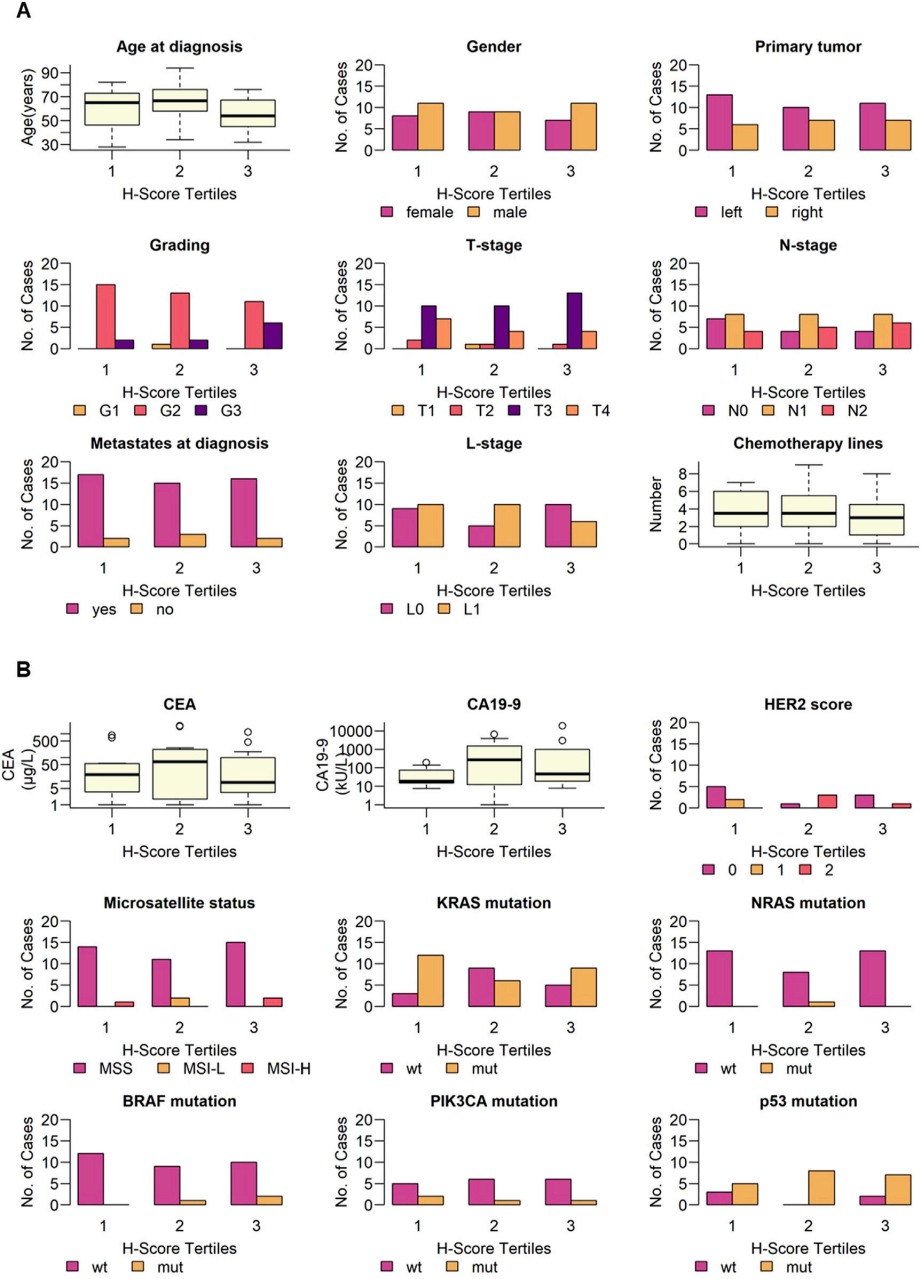
Clinical and pathological parameters and B7-H3 expression. B7-H3 expression (H-Score) was grouped in equally sized tertiles (1 = 0–190, 2 = 190–285, 3 = 285–300). Indicated **(A)** demographic parameters, disease status, number of chemotherapy lines and **(B)** tumor markers, and molecular and genetic alterations are shown for low, intermediate and high B7-H3 expression, respectively. Categorical variables are shown as bar plots, continuous variables are shown as boxplots.

To analyze difference in clinical and pathological patient parameters depending on B7-H3 expression, patients were subgrouped in three tertiles by H-Score (1 = 0–190, 2 = 190–285, 3 = 285–300). No significant difference for age at diagnosis and gender was observed (p = 0.36 and p = 0.84, respectively). Next, parameters for the disease status at diagnosis were analyzed for differences regarding B7-H3 expression levels. No significant differences for these parameters were observed: primary tumor localization (p = 0.83), tumor grading (p = 0.23), T-stage (p = 0.80), N-stage (p = 0.87), metastases at diagnosis (p = 0.89), and lymphovascular invasion (L-stage) (p = 0.31). Furthermore, we investigated if the treatment has an impact on B7-H3 expression. The number of chemotherapy lines (p = 0.60) did not show any significant effect on tumor-B7-H3 expression (Figure 2A). In addition to patient characteristics, the clinical parameters of tumor markers (soluble, tumor-expressed and mutations) were associated with H-Score tertiles. For serum CEA level (p = 0.41), CA19-9 level (p = 0.81), HER2 score (p = 0.05), microsatellite status (p = 0.22), KRAS mutation (p = 0.09), NRAS mutation (p = 0.26), BRAF mutation (p = 0.49), PIK3CA mutation (p = 1.00), and p53 mutation (p = 0.22), no significant differences were observed between the three H-Score tertiles (Figure 2B).

In summary, no significant association was found between B7-H3 expression, stratified by H-score tertiles, and the clinical or pathological characteristics of patients with mCRC.

### Patient outcome and B7-H3 H-Score

**FIGURE 3:**
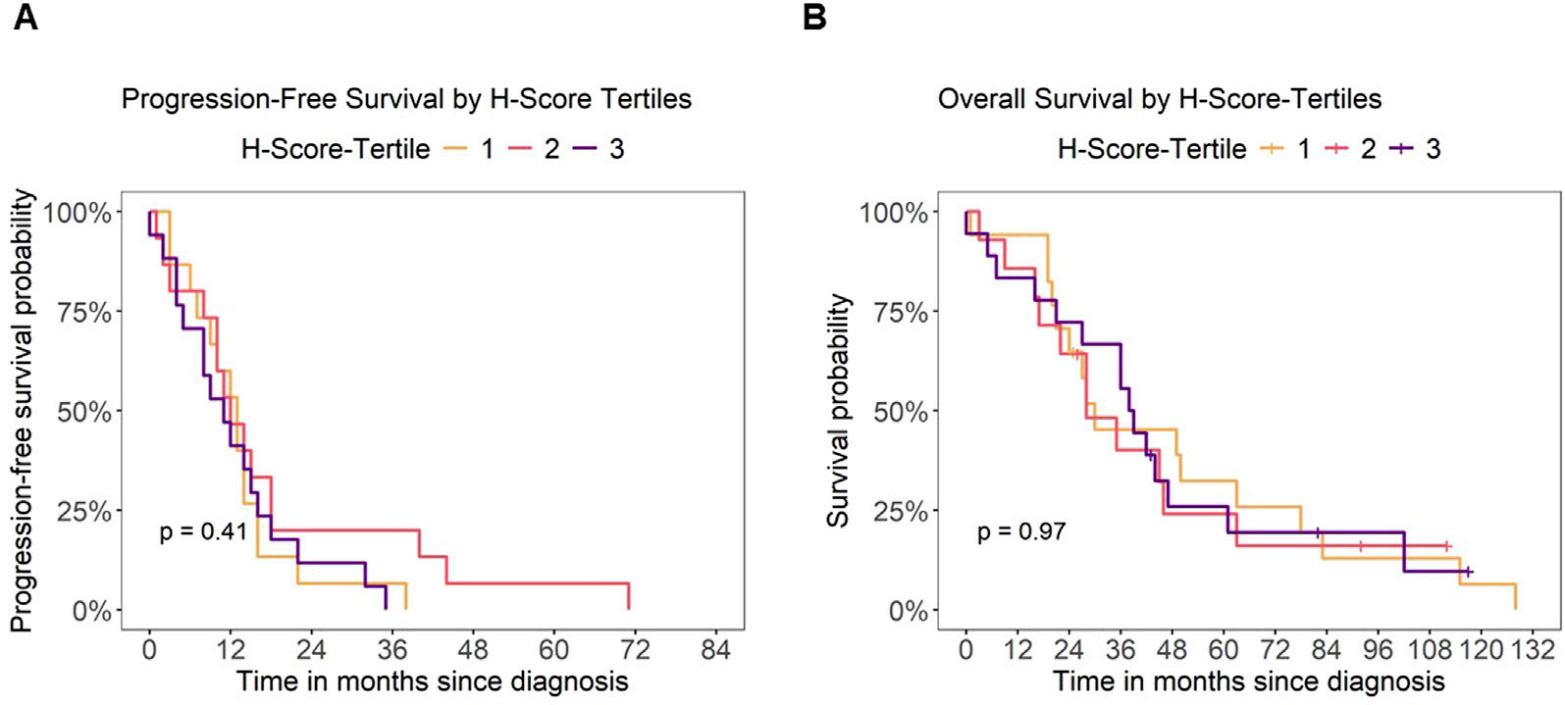
Patient outcome and B7-H3 expression. Analysis of outcome for mCRC patients depending on B7-H3 expression (H-Score tertiles). Kaplan-Meier analysis of **(A)** progression-free survival (PFS) and **(B)** overall survival (OS) by H-Score tertiles, respectively. Censoring for overall survival is indicated by a “+” symbol.

Next, we investigated if expression levels of B7-H3 have a potential impact on the outcomes of mCRC patients. To this end, we analyzed the association of B7-H3 H-scores with PFS and OS. A logrank test showed no significant difference in PFS across the three H-Score groups (p = 0.41), indicating that B7-H3 expression was not associated with PFS in this cohort (Figure 3A). Similarly, no significant differences in OS were observed between the H-Score tertiles (p = 0.97) (Figure 3B).

### Longitudinal analysis of B7-H3 expression

**FIGURE 4:**
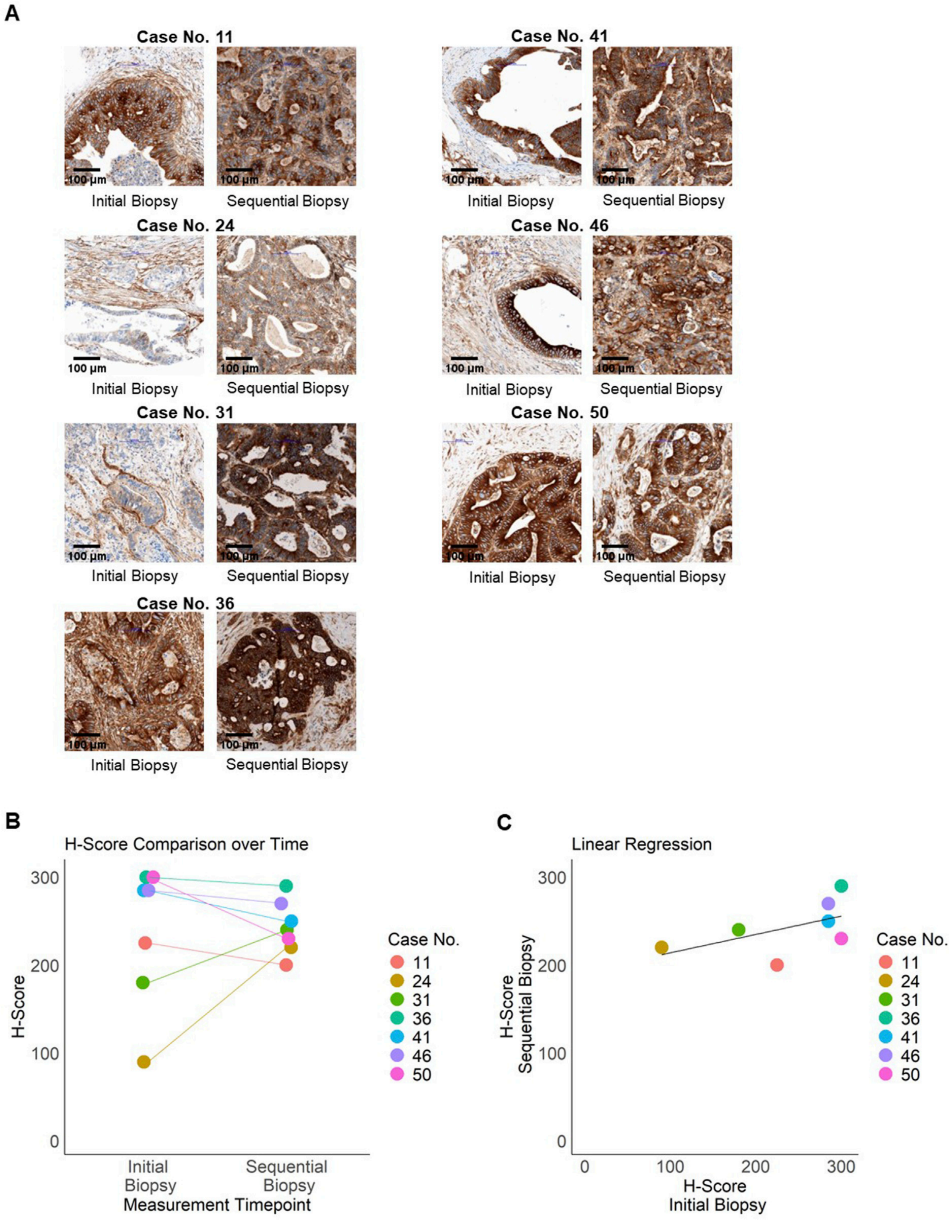
Longitudinal expression of B7-H3. Expression of B7-H3 in sequential patient samples (n = 7) from metastatic resections was assessed by immunohistochemistry. **(A)** Representative images of paired samples for B7-H3 staining are shown. Left panel: initial staining (TMA), right panel: sequential staining for the same patient. Bar scale 100 µm. **(B)** Initial and sequential H-Scores per patient (case numbers indicated) are illustrated in a jitter plot. **(C)** Regression analysis of initial and sequential H-Scores, with each data point representing an individual patient, color-coded by case number. The black line indicates the regression line.

Finally, we investigated whether alterations of B7-H3 expression levels in tumor tissues of mCRC patients occurred after the progression of the disease. Therefore, longitudinal, sequential data from an additional biopsy were immunohistochemically analyzed from n = 7 patients. The mean time between the two biopsies was 19.71 months (SD = 6.55), with a range from 7 to 27 months. The analysis focused exclusively on metastatic lesions, considering sequential, subsequent specimens from the liver (n = 6) and the lung (n = 1) (Figure 4A). The range of B7-H3 H-scores was found to vary from 90 to 300 at the initial analysis, and from 200 to 290 for the sequential biopsies time (Figure 4B). It should be noted that the initial values represent the mean scores from two replicates, whereas the sequential biopsies were based on a single sample per patient. No significant difference in H-Scores was found between the two time points (paired Wilcoxon test, p = 0.81). H-Scores at both time points showed a moderate positive correlation (Spearman’s ρ = 0.56). Although not statistically significant (p = 0.19), the moderate correlation suggests a potential trend toward consistent B7-H3 expression over time (Figure 4C).

These results demonstrate that B7-H3 is expressed in mCRC on both primary tumor tissue and metastases and is not altered during progression of the disease.

## Discussion

CRC, particularly in its metastatic stage, remains challenging to treat and is associated with poor survival outcomes. Immunotherapy using ICIs has shown clinical benefit, but its efficacy is largely restricted to the MSI-high/dMMR subgroup, which represents only a minority of mCRC patients [28]. Beyond checkpoint inhibition, the search for relevant tumor-associated target antigens is crucial for expanding therapeutic options. Among these, HER2 has emerged as a potential target in approximately 3%–5% of mCRC patients, offering targeted therapy options for this limited subset [29]. Nevertheless, the majority of mCRC patients lack effective targeted treatments, highlighting the urgent need for novel immunotherapeutic strategies aimed at additional tumor antigens. Addressing this challenge requires not only targeting the tumor cells directly but also modulating the tumor microenvironment to improve treatment responses. B7-H3 has gained attention as a promising therapeutic target due to its involvement in tumor progression, immune regulation, and its expression on both tumor cells and vasculature [30, 31]. In line with its immunoregulatory function, B7-H3 is also expressed beyond malignant epithelial cells, including on stromal fibroblasts and tumor-associated endothelial cells, underscoring its broader involvement within the tumor microenvironment. Studies in colorectal, ovarian, and pancreatic cancers have suggested that stromal and vascular expression of B7-H3 may be associated with immune modulation and, in some contexts, with poorer survival outcomes [12, 32–35]. These findings further support the rationale for targeting B7-H3 not only on tumor cells but also within the tumor stroma and vasculature to enhance therapeutic efficacy. However, its precise role in antitumor immunity remains debated. Moreover, as a member of the B7 family of immunoregulatory molecules, B7-H3 is also expressed on various immune cells, including macrophages, monocytes, dendritic cells, myeloid-derived suppressor cells (MDSCs), and some T cell subsets, where it contributes to immune evasion and tumor progression by modulating the local immune landscape within the tumor microenvironment [36, 37].

In this study, we demonstrated that B7-H3 is consistently overexpressed in mCRC, independent of demographic factors such as gender and age. Furthermore, no significant differences in B7-H3 expression were observed for all investigated parameters including tumor localization, disease grading, or TNM staging. Notably, we did not observe a significant prognostic effect of T-stage within our cohort. This may be attributed to the uniformly metastatic status (M1) of all included patients, in which the presence of distant metastases likely exerts a dominant influence on survival, potentially overshadowing the prognostic contribution of the primary tumor’s T-stage. Additionally, no significant association was observed between B7-H3 expression levels and the number of chemotherapy lines administered to the patients. Consistently, the soluble serum tumor markers, the HER2 expression as well as other common genetic and molecular markers (MSI, KRAS, NRAS, BRAF, PIK3CA, p53) did not show a significant difference between B7-H3 expression levels. In light of these findings, it can be concluded that, in the context of the mCRC patient cohort under consideration, B7-H3 expression is universally present, irrespective of clinical and pathological characteristics. Our findings are in line with the work of others, who reported B7-H3 overexpression in CRC regardless of MSI/MSS status and without correlation to clinicopathological features for both CRC and locally advanced rectal cancer [38, 39]. In contrast, other therapeutically relevant markers exhibit more heterogeneity. For instance, a discordance in HER2 expression between primary tumors and liver metastases of the same patients has been demonstrated, which may limit the efficacy of targeted therapies in these cases [40].

In other tumor entities, such as clear cell renal cell carcinoma [41] and prostate cancer [42], studies have demonstrated that a higher B7-H3 expression is associated with poorer clinical outcomes. Previous studies in CRC have also suggested a prognostic role for B7-H3 [39, 43], however we did not observe a significant impact on clinical outcomes in our cohort. Conversely, another study observed minimal B7-H3 expression in CRC, yet associated low B7-H3/PD-L1 levels as indicative of better prognosis [44]. This discrepancy may stem from differences in cohort composition, tumor biology, or methodological approaches. Further prospective, large-scale studies are needed to clarify its prognostic significance in this setting. Additionally, one study has demonstrated that soluble B7-H3 levels are elevated in CRC patients, but decrease with disease progression and are associated with a worse prognosis [45]. Since our study focused on tumor cell/tissue expression, further research is required to link soluble and tumor B7-H3 levels within the same patient cohort. While we concentrated on membranous B7-H3 expression due to its relevance for tumor cell surface-targeted immunotherapies, previous reports have also described cytoplasmic and nuclear localization [8, 32], which may carry distinct prognostic implications and warrant further investigation.

A key aspect of our study is the longitudinal assessment of B7-H3 expression in sequential metastatic biopsies, which confirmed the stability or even elevated expression of B7-H3 over time. The absence of significant changes (loss or downregulation) suggests that B7-H3 remains a viable target throughout disease progression, supporting its potential for sustained therapeutic intervention. Additionally, prior chemotherapy did not alter B7-H3 expression, reinforcing the notion that B7-H3-targeted therapies could be applicable regardless of prior treatment history.

Given its stable and strong expression, B7-H3 represents an attractive target for immunotherapies, including bsAbs such as CC-3 (B7-H3xCD3), currently under clinical evaluation for treatment of CRC, breast cancer, sarcoma and penile carcinoma (NCT05999396) [19]. Importantly, our findings suggest that routine pre-treatment assessment of B7-H3 expression may not be necessary, potentially streamlining patient selection for B7-H3-directed therapies in CRC. Future research should focus on validating these findings in larger cohorts and exploring strategies to enhance therapeutic efficacy, particularly in the context of immune-modulating agents.

In conclusion, our study reinforces B7-H3 as a robust target for immunotherapy in mCRC, with consistent expression across diverse patient subgroups and disease stages, independent of demographic, pathological, and molecular characteristics. The ongoing clinical evaluation of B7-H3-targeted therapies may offer new hope for patients with advanced CRC, a population in urgent need of effective treatment options.

## Data Availability

The raw data supporting the conclusions of this article will be made available by the authors, without undue reservation.

## Abbreviations

bsAb, bispecific antibody; BRAF, B-Raf Proto-Oncogene, Serine/Threonine Kinase; CA19-9, carbohydrate-antigen 19-9; CAR, chimeric antigen receptor; CD, cluster of differentiation; CEA, carcinoembryonic antigen; CRC/mCRC, colorectal cancer/metastatic colorectal cancer; dMMR, deficient mismatch repair; FDA, U.S. Food and Drug Administration; FFPE, formalin-fixed, paraffin-embedded; H&E, hematoxylin and eosin; HER2, Human Epidermal Growth Factor Receptor 2; H-Score, Histoscore; ICI, immune checkpoint inhibitor; IQR, interquartile range; KRAS, Kirsten Rat Sarcoma Viral Oncogene Homolog; MDSCs, myeloid-derived suppressor cells; MSI/MSS, microsatellite instability/microsatellite stability; MSI-H/MSI-L, microsatellite instability-high/-low; NRAS, Neuroblastoma RAS Viral Oncogene Homolog; NRG1+, neuregulin 1-positive; NCT, national clinical trial number; OS, overall survival; p53, tumor protein 53; PIK3CA, Phosphoinositide-3-Kinase Catalytic Subunit Alpha; PFS, progression-free survival; SD, standard deviation; TMA, tissue microarray; UICC, Union Internationale Contre le Cancer; WSI, whole slide imaging.
