# Supplementary material for: B7-H3: a consistent marker in metastatic colorectal cancer with potential for targeted treatment
Source: Pathol Oncol Res. 2025 Aug 13;31:1612186. doi: 10.3389/pore.2025.1612186 (PMC12380622; doi:10.3389/pore.2025.1612186)
Supplement: Supplementary file 1 [file DataSheet1.pdf]

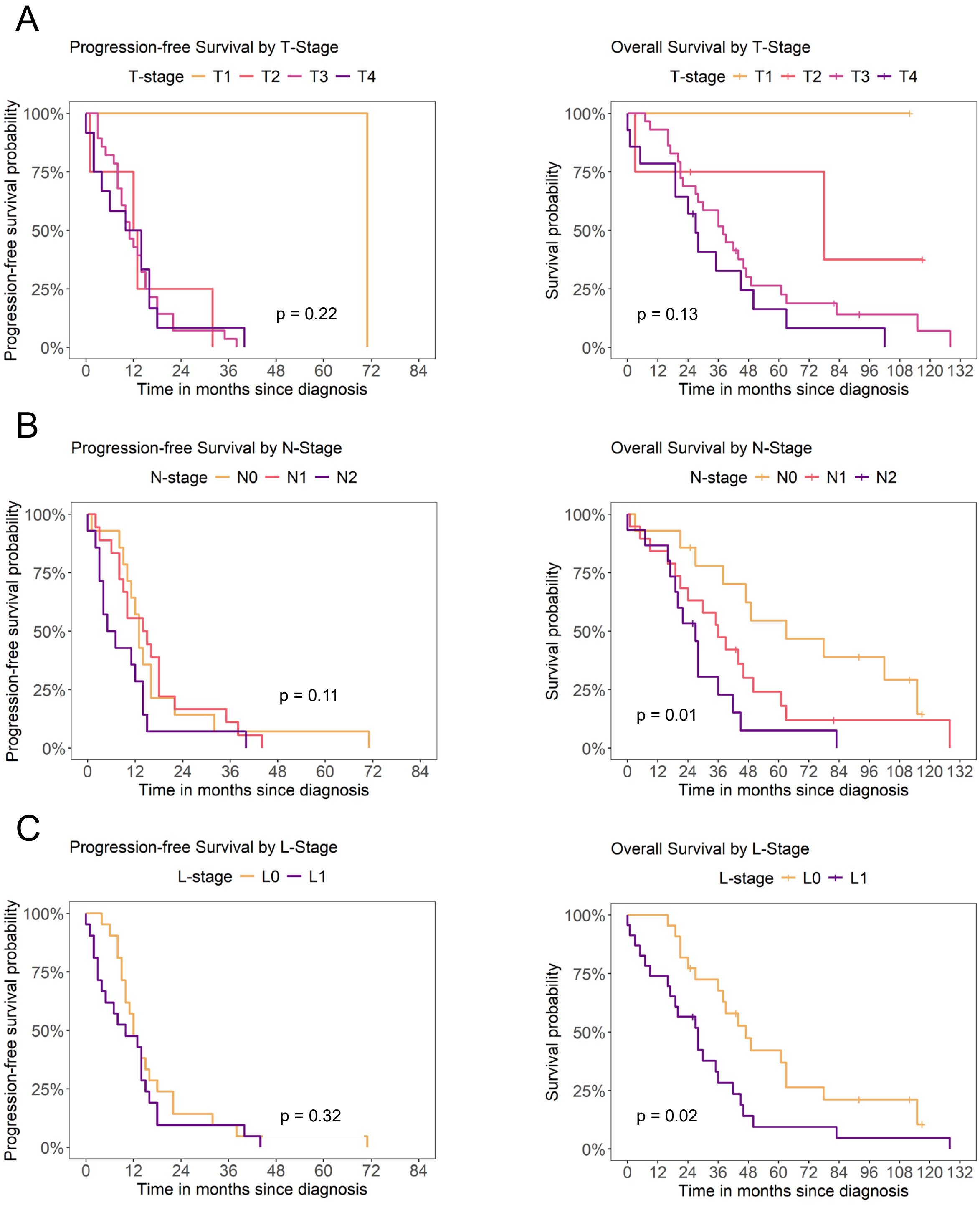

Supplementary Figure 1: Survival plots by staging.

Kaplan-Meier plots of progression-free survival (PFS) and overall survival (OS) according to **A** T-stage (depth of invasion of the primary tumor), **B** N-stage (nodal metastasis) **C** L-stage (lymphatic invasion). Censoring for overall survival is indicated by a "+" symbol.

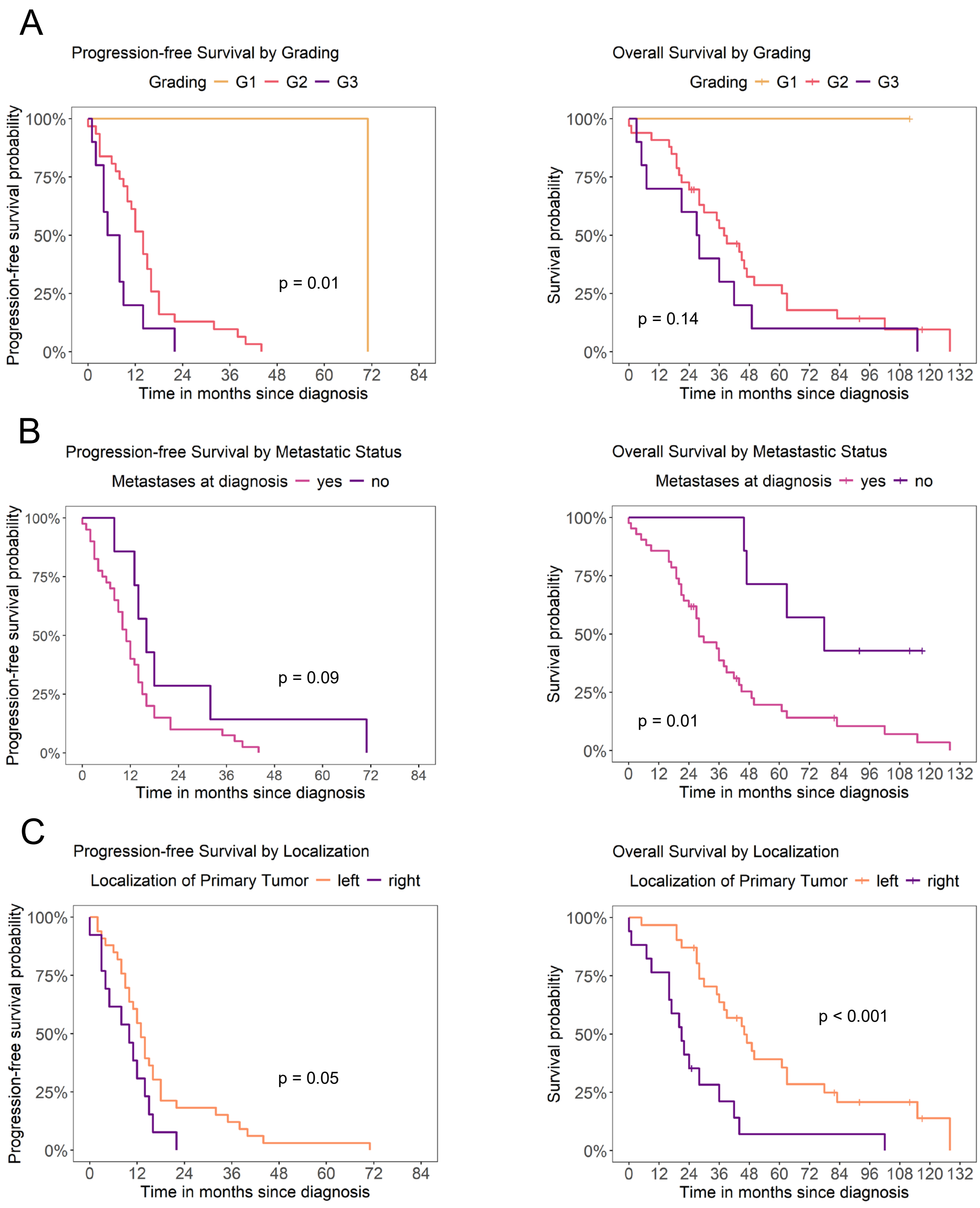

Supplementary Figure 2: Survival plots by grading, metastatic status and localization of primary tumor.

Kaplan-Meier plots of progression-free survival (PFS) and overall survival (OS) according to **A** grading, **B** metastatic status and **C** localization of primary tumor. Censoring for overall survival is indicated by a "+" symbol.

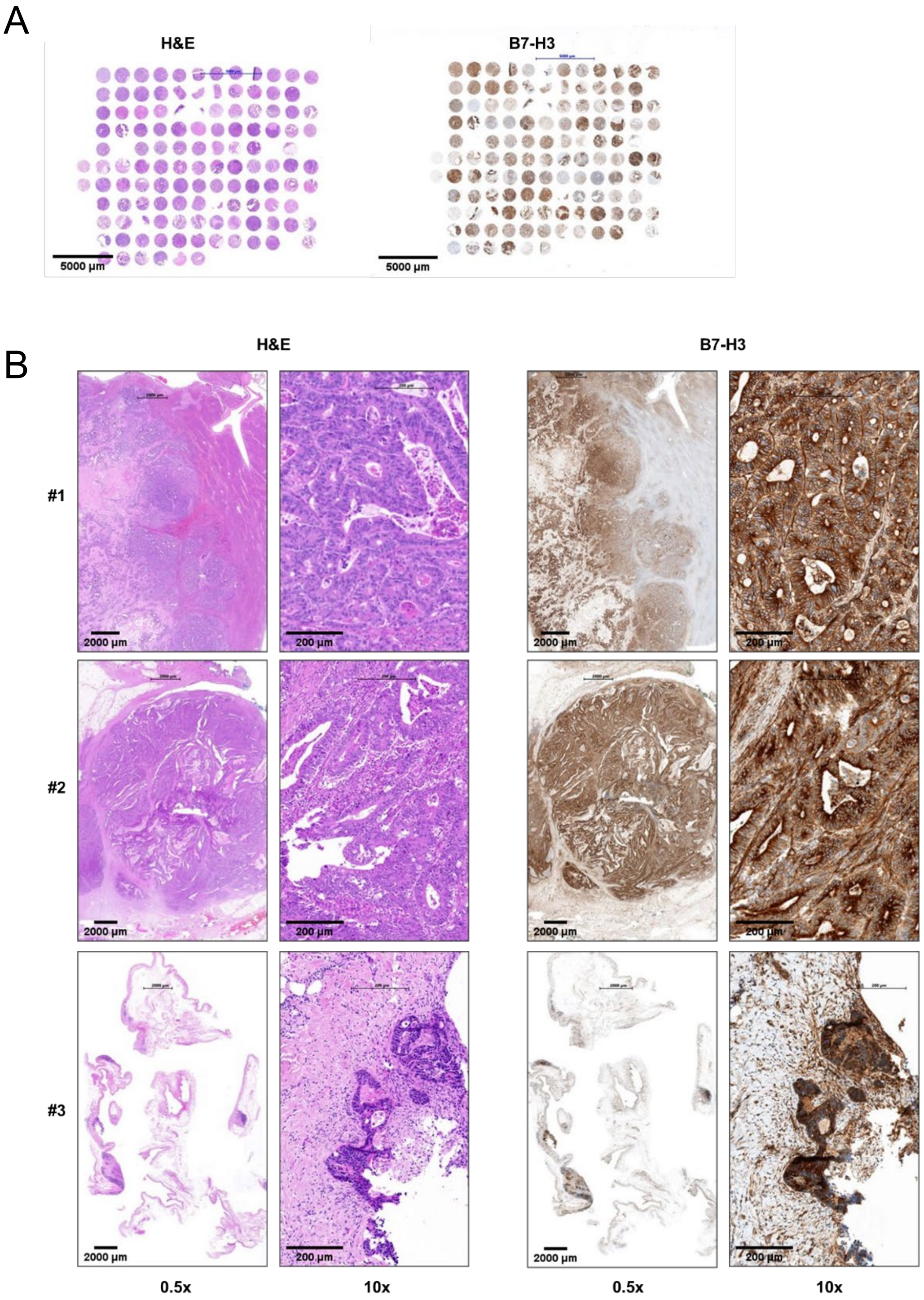

Supplementary Figure 3: Staining of B7-H3 on paraffine-embedded tissues from colorectal cancer patients

Freshly cut colorectal carcinoma tissues were formalin-fixed and paraffin-embedded and stained for B7-H3 by immunohistochemistry. **A** Overview images of sections of the created tissue microarray (TMA) for hematoxylin and eosin (H&E) (left panel) and B7-H3 (right panel) staining are shown. Bar scale indicates 5000 µm. **B** Exemplary images of whole slide sections of primary tumor or metastases resections are shown. Left panels depict H&E staining and right panels depict B7-H3 staining with either 0.5x magnification (bar scale 2000 µm) or 10x magnification (bar scale 200 µm) as indicated.
